# Impact of laryngeal sequelae on voice- and swallowing-related outcomes in paracoccidioidomycosis

**DOI:** 10.1590/1678-9199-JVATITD-2020-0008

**Published:** 2020-08-17

**Authors:** Neisa Santos Carvalho Alves Pissurno, Lucas da Motta Esteves, Juliana Marques Benedito, Vanessa Ponsano Giglio, Lídia Raquel de Carvalho, Rinaldo Poncio Mendes, Anamaria Mello Miranda Paniago

**Affiliations:** 1 Medical School, Federal University of Mato Grosso do Sul (UFMS), Campo Grande, MS, Brazil.; 2 Maria Aparecida Pedrossian University Hospital, Federal University of Mato Grosso do Sul (UFMS), Campo Grande, MS, Brazil.; 3 Botucatu Biosciences Institute (IBB), São Paulo State University (UNESP), Botucatu, SP, Brazil.; 4 Botucatu Medical School, São Paulo State University (UNESP), Botucatu, SP, Brazil.

**Keywords:** Paracoccidioidomycosis, Voice, Larynx, Quality of life, Deglutition

## Abstract

**Background:**

The present study was carried out aiming to evaluate the impact of laryngeal sequelae on the quality of life of treated paracoccidioidomycosis (PCM) patients.

**Methods:**

This cross-sectional study was conducted at the Otorhinolaryngology Outpatient Clinic of the University Hospital, Federal University of Mato Grosso do Sul, Brazil. Thirty-two PCM patients considered clinically and immunologically cured were included: 16 with laryngeal involvement during the active phase of the disease (laryngeal PCM group) and 16 without laryngeal involvement (control group). They were submitted to structured interview, otorhinolaryngology examination, videolaryngoscopy, videoendoscopic swallowing study, completed two questionnaires for voice self-assessment - Voice-related Quality of Life (V-RQOL) and Voice Handicap Index (VHI) - and were asked to score their voices on a scale from zero to 10 (self-assessment of vocal quality).

**Results:**

Dysphonia was present in 50% of the cases. Patients with laryngeal PCM presented worse voice-related quality of life scores on the V-RQOL and poorer vocal quality self-assessment than the control group. No significant differences in the VHI were found between the groups. None of the participants developed dysphagic sequelae, although some minor changes were observed on videoendoscopic examination.

**Conclusion:**

There were no dysphagia complaints and only a few mild changes were found on the fiberoptic endoscopic evaluation of swallowing, suggesting that this evaluation should be performed only in specific cases. Patients with laryngeal involvement presented worse V-RQOL and self-assessment voice quality. This study contributes to the current knowledge of the functional assessment of the larynx affected by PCM and the impact of dysphonia on quality of life.

## Background

Paracoccidioidomycosis (PCM) is a systemic and endemic mycosis restricted to Latin America caused by fungi of the genus *Paracoccidioides.* Laryngeal involvement is found in 22 to 43% of cases with the chronic form of the disease, which can lead to irreversible lesions [1, 2].

Dysphonia is the main functional sequelae of the larynx and usually significantly impairs quality of life [2]. This impact on personal, social and professional relationships is not necessarily directly related to the degree of dysphonia; therefore, the use of self-assessment voice quality instruments in the management of dysphonia has become increasingly valued, especially to assess therapeutic responses, sequelae and rehabilitation [3, 4, 5]. 

Dysphagia is another functional sequelae that can occur in patients with PCM with laryngeal involvement and should be investigated since swallowing disorders can lead to respiratory infections, which greatly contribute to the morbidity and mortality of PCM as most PCM patients already exhibit pulmonary involvement.

The lack of studies on laryngeal sequelae, especially regarding the quality of life of patients with PCM, motivated this study, the objective of which was to evaluate anatomical and functional laryngeal sequelae and their impact on patients’ quality of life.

## Methods

### Patients

This cross-sectional study was conducted at the Otorhinolaryngology Outpatient Clinic of the University Hospital, Federal University of Mato Grosso do Sul, between September 2016 and April 2017 in Campo Grande, Mato Grosso do Sul, Brazil. The hospital’s Systemic Mycoses Center has registered all demographic and clinical information of PCM patients seen at the center in a database since 2000. The study was approved by the institutional Research Ethics Committee under protocol no. 1,705,604 (August 31, 2016).

*Inclusion criteria* were:

- Patients aged 18 years or older.
- Patients diagnosed with the chronic form of PCM as confirmed by identification of the typical forms of *Paracoccidioides* spp. in clinical material by direct mycological or histopathological examination, or by identification of specific antibodies by the double agar gel immunodiffusion (DID) test.
- Patients considered clinically cured based on symptom resolution and immunologically cured as defined by negative serology on the DID test for at least one year.

The *exclusion criteria* included patients with anatomical and/or functional changes in the upper aerodigestive pathway due to other etiologies.

**Figure 1.**
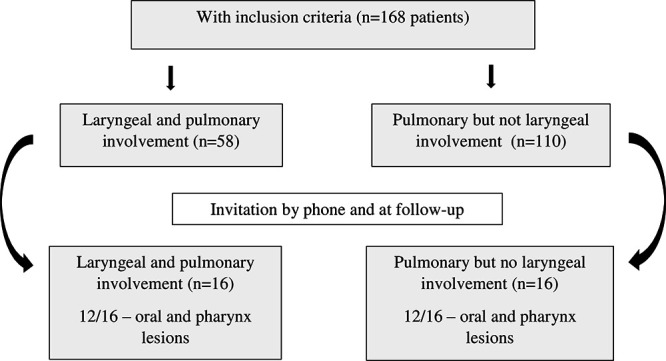
Flowchart of paracoccidioidomycosis patients evaluated regarding voice- and swallowing-related quality of life.

A total of 168 patients in the database met the above criteria, and all had pulmonary involvement during the active phase of the disease. Of these, 58 also had concomitant laryngeal involvement at the time of PCM diagnosis and were contacted by phone or in person during routine consultation and invited to participate in the study. Sixteen patients accepted the invitation and constituted the PCM-laryngeal group. In this group, 12 patients had concomitant lesions in the oral cavity and/or oropharynx at the time of diagnosis. The 110 patients without laryngeal involvement on admission were also considered for participation in the study. The first 16 patients who accepted the invitation constituted the control group. In this group, 12 patients also had concomitant lesions in the oral cavity and/or oropharynx at the time of diagnosis. The control group was recruited strategically to avoid bias in the analysis of laryngeal function and deglutition (Figure 1).

### Procedures

*Demographic and clinical data*

Demographic and clinical data were obtained by the researcher from structured interviews with the study participants and from their medical records using a form previously prepared for this purpose.

*Ear-nose-throat examination, videolaryngoscopy and fiberoptic endoscopic evaluation of swallowing*

The ear-nose-throat (ENT) examination and fiberoptic endoscopic evaluation of swallowing (FEES) of all patients was performed by the same otorhinolaryngologist (NSCAP), with the assistance of a speech therapist (VPG) - who recorded the oroscopic, videolaryngoscopic and swallowing findings on a standardized form. The videoendoscopic device used included a Karl-Storz® camera with an endocoupler, a Karl-Storz® xenon light source, a Sony® Trinitron monitor and a Karl-Storz® 8-mm rigid laryngoscope with 70 degrees of angulation for videolaryngoscopic and Pentax® 3.4-mm nasal fiberscope coupled to the Karl-Storz® for FEES. 

The alterations observed on videolaryngoscopy were classified as to severity by a score based on its occurrence, sites involved, and type of lesions (VLS-score), as follows: 0 - no alteration; 1 - edema or hyperemia anywhere; 2 - amputation and/or synechia in the supraglottic region; 3 - amputation and/or synechia in the glottic region.

Foods with a liquid, thin and pasty, thick and pasty and solid consistency were offered. Filtered water stained with blue food dye served as the item with a liquid consistency and was offered to the participants in the form of small sips from a disposable plastic cup. The foods with thick and thin pasty consistencies were prepared using a starch-based commercial thickener (according to the manufacturer's instructions) dissolved in filtered water and stained with blue food dye, which were provided on disposable 5-mL plastic spoons. Water and salty crackers stained with blue food dye were used for the food with a solid consistency, which was placed directly in each individual's mouth.

Nasal regurgitation, delayed swallowing, posterior escape, stasis or laryngotracheal penetration/aspiration were the parameters analyzed during FEES.

Swallowing alterations were classified regarding severity by a rating proposed by the examiner, as follows:

- *Mild*: impairment manifested in some swallows without causing discomfort to the patient that is easily circumvented with facilitating maneuvers, such as coughing and throat clearing.
- *Moderate*: impairment observed in all swallows causing some discomfort to the patient, such as choking and a feeling of suffocation; however, it still responsive to facilitating maneuvers.
- *Severe*: evident impairment causing substantial discomfort to the patient that is not reversible with facilitating maneuvers and can potentially lead to immediate clinical repercussions, such as laryngotracheal or posterior aspiration and aspiration pneumonia.

In this study, the examiner considered the symptom of dysphagia to be present in patients with a persistent complaint of difficulty or discomfort in swallowing of one or more food consistencies, leading to consistency restriction or the need for facilitating maneuvers for symptom relief. The presence of occasional swallowing complaints or the occasional use of facilitating maneuvers, such as coughing and throat clearing, was not considered a dysphagic symptom.

*Voice analysis*

All patients completed two self-assessment instruments validated for the Portuguese language:

- The Voice-Related Quality of Life (V-RQOL) measure adapted from the American version [6] into the Portuguese language [7], which consists of ten questions covering physical and socioemotional domains, with scores ranging from 0 to 100 for each domain.
- The Voice Handicap Index (VHI) adapted from the American version [8] into the Portuguese language [9], which consists of 30 questions addressing functional, physical and emotional domains, with scores ranging from 0 to 40 for each domain.

For the comparative analysis of the questionnaire results, the weighted means of the total scores and the scores for each domain were calculated for both instruments, which varied from 0 to 100. For the V-RQOL, a value closer to 100 corresponds to better voice- related quality of life, while the inverse pattern applies to the VHI. 

The voice analysis was complemented by a self-assessment of voice quality defined by an integer number between 0, which was associated with a voice considered poor by the patient, and 10, which was associated with a voice considered excellent. The voice quality estimated by the patient was classified into ranges as follows: 0 to 3, poor quality; 4 to 6, moderate quality; and 7 to 10, high quality.

The presence of chronic voice complaints since PCM diagnosis/treatment and/or a voice change diagnosed by the auditory-perceptual analysis performed by the otorhinolaryngologist or speech therapist was considered dysphonic sequelae; other causes of voice changes were not considered. 

*Data processing and statistical analysis*

The data collected were entered in Microsoft Excel 2010, allowing the creation of reports according to the variables of interest.

Prevalence rates in the groups were evaluated by performing the test for comparison of two proportions using the normal distribution. Fisher’s exact test was used to analyze categorical data in 2X2 contingency tables. Comparisons of three or more dichotomous variables in dependent samples were carried out using the Cochran Q test. The central measures of two populations were compared by the Mann-Whitney test. Correlations between variables were evaluated by Spearman’s correlation coefficient. Significance was set at 5% and the software used was SPSS version 21. 

## Results

The 32 study patients had a mean age of 51.3 ± 7.6 years at the time of diagnosis and 60.6 ± 8.4 years at the time of this study. Table 1 shows that the study groups did not differ in demographic or clinical data. 

**Table 1.** Baseline characteristics of the paracoccidioidomycosis patients with (n = 16) and without (n = 16) laryngeal involvement.

Characteristics	Laryngeal involvement		p value*
	No (n =16) n (%)	Yes (n = 16) n (%)	
**Male gender**	16 (100)	15 (93.8)	0.50
**Ethnicity**			
White	4 (25.0)	5 (31.3)	0.28
Mixed/Black	12 (75.0)	11 (68.7)	
**Residence in a rural zone**			
Previous	16 (100)	16 (100)	1.00
Current	6 (37.5)	3 (18.8)	0.27
**Smoker (current or previous)**	16 (100)	15 (93.8)	0.50
**Alcohol intake (current or previous)**	16 (100)	15 (93.8)	0.50
**Clinical severity**			
Mild	1 (6.3)	1 (6.3)	0.10
Moderate	12 (75.0)	8 (50.0)	
Severe	3 (18.7)	7 (43.7)	
**Antifungal compound**			
Trimethoprim-sulfamethoxazole	12 (75.0)	13 (81.3)	0.64
Itraconazole	3 (18.7)	3 (18.7)	
Ketoconazole	1 (6.3)	-	

*Fisher's exact test.

All patients had different degrees of pulmonary sequelae, i.e., pulmonary fibrosis, identified by simple chest radiography and/or computed tomography. The prevalence of anatomical and/or functional sequelae in the larynx in the PCM-laryngeal group was 56.3% (9 patients), with 37.5% (6 patients) of these patients exhibiting anatomical sequelae - without predominance in the anatomical site (p = 0.91) or lesion type (p = 0.57) - 50% (8 patients) exhibiting dysphonia and no patient with dysphagia. 

The following morphological aspects and lesion sites were observed at videolaryngoscopy: amputation of the epiglottis (n = 3), synechia of the epiglottis (n = 1), synechia of the interarytenoid fold (n = 1) and edema and/or hyperemia of the vocal fold (n = 2).

No correlation was observed between VLS-score and the three variables analyzed: VLS-score and V-RQOL-T: (r_s_ = 0.062; p = 0.82); VLS score and VHI-T: (r_s_ = -0.003, p = 0.99); and VLS score and SE: (r_s_ = 0.310; p = 0.24), by Spearman correlation.

On the V-RQOL questionnaire, the PCM-laryngeal group had worse (lower) scores for the physical domain questions, in contrast, the control group present no predominance of any domain. For the VHI, no significant differences were observed in the scores between the groups or predominance in any domain (Table 2).

 In the self-assessment of voice quality, 14 (87.5%) patients in the PCM-laryngeal group reported high voice quality scores, while only one patient reported poor voice quality and one patient reported moderate voice quality. All the patients in the control group reported high voice quality, assigning scores equal to or greater than 7 (Table 2).

**Table 2.** Comparison of the vocal analyses performed by three different methods in the treated paracoccidioidomycosis patients with (16 cases) and without (16 cases) laryngeal involvement.

	Laryngeal involvement		p value*
	No (n = 16)	Yes (n = 16)	
	Med [IQR]	Med [IQR]	
**V-RQOL**			
Physical	91.7 [87.5; 100.0]	79.2 [58.3; 89.6]	0.02
Socioemotional	100.0 [90.6; 100.0]	100.0 [87.5 - 100.0]	0.57
**Total**	95.0 [88.8; 100.0]	85.0 [68.8; 92.5]	0.04
**VHI**			
Emotional	0.0 [0.0; 3.5]	2.0 (0.0; 5.5)	0.32
Functional	1.0 [0.0; 10.5]	5.0 [2.0; 10.0]	0.19
Physical	4.0 [0.5; 11.5]	12.0 [4.5; 14.0]	0.09
**Total**	7.0 [2.5; 23.5]	18.0 [10.5; 27.0]	0.07
**Self-assessment**	9.5 [8.5; 10.0]	8.0 [7.0; 9.0]	0.02

*Mann-Whitney test

Med: median; IQR: interquartile range; V-RQOL: Voice-Related Quality of Life Measure [7]; VHI: Vocal Handicap Index [9]; Self-assessment: score from zero to ten assigned by the patient to his or her voice quality.

The control and PCM-laryngeal groups had similar correlations when the V-RQOL variables were analyzed among themselves, when the VHI variables were analyzed among themselves, and when the V-RQOL and VHI variables were analyzed together (Table 3). However, the two groups differed substantially in the correlations between self-assessment voice-related quality of life and the different variables of the V-RQOL and VHI. In the PCM-laryngeal group, all these correlations were significant, whereas in the control group, self-assessment voice quality was correlated only with the physical domain of the V-RQOL measure (V-RQOL-P).

**Table 3.** Correlations among the V-RQOL and VHI questionnaires and self-assessment voice quality in the 32 patients with residual paracoccidioidomycosis (PCM), including 16 with (PCM-laryngeal) and 16 without (control) laryngeal involvement.

PCM without laryngeal involvement (n = 16)								
		V-RQOL-P	V- RQOL-SE	V-RQOL-T	VHI-E	VHI-F	VHI-P	VHI-T
**V-RQOL-SE**	r	0.727	-	-	-	-	-	-
	p	0.001						
**V-RQOL-T**	r	0.946	0.905	-	-	-	-	-
	p	0.000	0.000					
**VHI-E**	r	-0.712	-0.750	-0.756	-	-	-	-
	p	0.002	0.001	0.001				
**VHI-F**	r	-0.756	-0.713	-0.785	0.756	-	-	-
	p	0.001	0.002	0.000	0.001			
**VHI-P**	r	-0.861	-0.868	-0.939	0.859	0.831	-	-
	p	0.000	0.000	0.000	0.000	0.000		
**VHI-T**	r	-0.835	-0.833	-0.900	0.920	0.927	0.962	-
	p	0.000	0.000	0.000	0.000	0.000	0.000	
**S-E**	r	0.662	0.191	0.474	-0.260	-0.363	-0.431	-0.383
	p	0.005	0.479	0.064	0.330	0.167	0.096	0.143
**PCM with laryngeal involvement (n = 16)**								
		**V-RQOL-P**	**V- RQOL-SE**	**V-RQOL-T**	**VHI-E**	**VHI-F**	**VHI-P**	**VHI-T**
**V-RQOL-SE**	r	0.583	-	-	-	-	-	-
	p	0.018						
**V-RQOL-T**	r	0.911	0.863	-	-	-	-	-
	P	0.000	0.000					
**VHI-E**	r	-0.644	-0.845	-0.824	-	-	-	-
	p	0.007	0.000	0.000				
**VHI-F**	r	-0.719	-0.694	-0.803	0.856	-	-	-
	p	0.002	0.003	0.000	0.000			
**VHI-P**	r	-0.789	-0.695	-0.851	0.778	0.699	-	-
	p	0.000	0.003	0.000	0.000	0.003		
**VHI-T**	r	-0.771	-0.807	-0.891	0.955	0.933	0.880	-
	p	0.000	0.000	0.000	0.000	0.000	0.000	
**S-A**	r	0.558	0.705	0.699	-0.848	-0.695	-0.712	-0.813
	p	0.025	0.002	0.003	0.000	0.003	0.002	0.000

V-RQOL: Voice-Related Quality of Life Measure; V-RQOL-P: V-RQOL physical domain; V-RQOL-SE: V-RQOL socioemotional domain; V-RQOL-T: V-RQOL total score; VHI: Vocal Handicap Index; VHI-E: VHI emotional domain; VHI-F: VHI functional domain; VHI-P: VHI physical domain; VHI-T: VHI total score. S-A: self-assessment voice quality; r: Pearson correlation coefficient; p*:* p value.

None of the 32 study patients developed dysphagia. Tooth loss was present in nine patients (56.3%) in the PCM-laryngeal group and in seven patients (43.8%) in the control group (0.70 < p < 0.80). Laryngeal sensitivity was altered in four patients (25.0%) in the PCM-laryngeal group and in two patients (12.5%) in the control group (p = 0.24).

Despite the absence of dysphagic sequelae in the study participants, some mild swallowing changes with no clinical repercussions were found on FEES in 50% of the subjects in both groups, with no predominance of any type of swallowing change or food consistency.

## Discussion

PCM is the most prevalent chronic infection of the upper airdigestive tract in Latin America, whose importance is related to its severity, sequelae in spite of appropriate therapy, and repercussion on the quality of life [10, 11]. Consistent with previous studies [12, 13, 14, 15], most of our patients were men, adults, cigarette smokers and alcoholics who previously resided or currently reside in rural areas.

The prevalence of PCM with laryngeal involvement ranges from 22 to 43% [1, 13], and dysphonia is the most commonly associated symptom, which is present in 50 [16] to 86% [17] of cases. In the present study, 34.5% of the patients with PCM had laryngeal involvement at the time of diagnosis, and dysphonia was the main symptom reported (81%). Notably, however, the absence of laryngeal symptoms does not exclude laryngeal involvement of the disease, which may be more prevalent than described [18].

Laryngeal sequelae due to PCM are important because of their frequency even after effective therapy and the severity with which they occur in many cases. Silva et al*.* [19] observed anatomical and functional sequelae in 40% of PCM cases with laryngeal involvement, and dysphonia was the main functional sequelae found. Our study revealed a higher prevalence of anatomic and/or functional laryngeal sequelae (56.3%). Although dysphonia is the most frequent functional sequelae in laryngeal PCM, its prevalence varies from 21% [20] to 50% [21]. This discrepancy is related to the patient selection criteria and the definition of dysphonia used.

No correlation between severity of the residual lesions evaluated by videolaryngoscopy (VLS-score) and any of the other methods for assessing voice-related quality of life was demonstrated. Da Costa et al. [22] observed that patients who had laryngeal involvement in the active phase of PCM remain with impaired voice after treatment, even without any fibrotic scar visible at laryngoscopy. Residual dysphonia may be due to the scarring process of the lesions or to functional adjustment mechanisms developed during the voice limitation phase [24]

The concept of a normal voice is subjective and varies widely. The voice may be considered normal when it sounds pleasant to the listener and when it is produced effortlessly with characteristics consistent with the speaker’s gender, age, body structure and personality [23]. Therefore, the degree of dysphonia does not always have a direct relationship with an individual's quality of life because the intensity of vocal limitation depends on social, emotional and even occupational factors [5, 24, 25].

Thus, the use of validated instruments to measure the impact of dysphonia on quality of life is increasing, especially in the evaluation of treatment responses and rehabilitation [4, 5, 24, 25]. Although the voice self-assessment instruments applied in this study showed strong correlations with one another, the V-RQOL and self-assessment voice quality results differentiated the patients with and without laryngeal involvement. The group with laryngeal involvement presented worse physical domain of V-RQOL and self-assessment voice-related quality of life scores than the control. This can be explained not only by the residual lesions in the larynx, but also by functional adjustment mechanisms developed during the voice limitation phase [22]. Thus, as the videolaryngoscopic evaluation underestimates the real sequelae, specific functional tests should be performed. None of the patients studied used their voices professionally, and all reported low voice demands and a low-quality level of vocal requirement, which may help explain this finding. Although widely used to evaluate voice professionals, especially teachers, the instruments to measure the impact of dysphonia on quality of life have not yet been applied in patients with PCM with laryngeal involvement, reflecting the originality of this study. The voice self-assessment instruments used were effective and simple to apply, facilitating their introduction into the routine follow-up of such patients.

The swallowing disorder and consequent impairment of the protective mechanisms of the airways may lead to laryngotracheal aspiration, resulting in respiratory disease ranging from bronchospasm to aspiration pneumonia, pulmonary abscess, sepsis and death [26]. Because of their potential severity, detection of swallowing disorders is extremely important, especially in patients with cardiopulmonary comorbidities.

FEES provides a rapid and effective evaluation with basic technical requirements and therefore constitutes a good screening test for oropharyngeal dysphagia, exhibiting results comparable to those found with videofluoroscopy, which is considered the gold standard examination [27, 28, 29, 30, 31, 32].

None of the patients in this study developed dysphagia, although some mild swallowing changes with no clinical repercussions were observed on FEES. The high prevalence of tooth loss and decreased laryngeal sensitivity on the swallowing evaluation, which are common in elderly individuals, may explain the presence of these changes on the videoendoscopic examination even in the absence of dysphagia and underlying anatomical sequelae.

This study presents some limitations. Although the number of patients seems to be small in relationship to other diseases, 168 patients were initially selected in the Service, to reach the32 ones that reached the criteria of inclusion, 16 of them for the study group and 16 for the control group. In addition, this number of patients permitted a statistical analysis of the results. Nevertheless, it could be very interesting the evaluation of a higher number of patients. It could be also interesting the evaluation of at least two more stages of the disease, between the active phase and the residual lesions. It could be suggested the evaluation just after clinical cure, usually early observed and at the serological cure, to have a sequential analysis.

## Conclusion

There were no dysphagia complaints and only a few mild changes were found on the FEES, suggesting that this evaluation should be performed only in specific cases. Patients with laryngeal involvement presented worse V-RQOL and self-assessment voice quality.

## References

[B1] Machado J, Miranda JL (1960). Considerations regarding South American blastomycosis - sites, symptoms, penetration pathways and dissemination in 313 consecutive cases. Hospital (RJ).

[B2] Machado J, Rego AP, Chaves ALF, Miranda JL, Silva CC (1960). Considerations regarding South American blastomycosis. Laryngeal and bronchial involvement in 104 cases: endoscopic findings. Hospital (RJ).

[B3] Leite APD, Carnevale LB, Rocha HL, Pereira CA, Lacerda L (2015). Relationship between voice self-assessment and clinical evaluation data in dysphonic individuals. Rev CEFAC.

[B4] Spina AL, Maunsell R, Sandalo K, Gusmão R, Crespo A (2009). Correlation between voice and life quality and occupation. Rev Bras Otorrinolaringol.

[B5] Tutya AS, Zambon F, Oliveira G, Behlau M (2011). Comparison of V-RQOL, VHI and VAPP scores in teachers. Rev Soc Bras Fonoaudiol.

[B6] Hogikyan ND, Sethuraman G (1999). Validation of an instrument to measure voice-related quality of life (V-RQOL). J Voice.

[B7] Gasparini G, Behlau M (2009). Quality of life: validation of the Brazilian version of the voice-related quality of life (V-RQOL) measure. J Voice.

[B8] Jacobson BH, Johnson A, Grywalski C, Silbergleit A, Jacobson G, Benninger MS (1997). The Voice Handicap Index (VHI): development and validation. Am J Speech Lang Pathol.

[B9] Santos LM, Gasparini G, Behlau M (2007). Validação do protocolo do Índice de Desvantagem Vocal (IDV) no Brasil.

[B10] Mendes RP, Cavalcante RS, Marques SA, Marques MEA, Venturini J, Sylvestre TF (2017). Paracoccidioidomycosis: current perspectives from Brazil. Open Microbiol J.

[B11] Shikanai-Yasuda MA, Mendes RP, Colombo AL, Queiroz-Telles F, Kono ASG, Paniago AMM (2017). Brazilian guidelines for the clinical management of paracoccidioidomycosis. Rev Soc Bras Med Trop.

[B12] Kamikawa CM, Mendes RP, Vicentini AP (2017). Standardization and validation of Dot-ELISA assay for Paracoccidioides brasiliensis antibody detection. J Venom Anim Toxins incl Trop Dis.

[B13] Paniago AMM, Aguiar JIA, Aguiar ES, Cunha RV, Pereira GROL, Londero AT (2003). Paracoccidioidomycosis: a clinical and epidemiological study of 422 cases observed in Mato Grosso do Sul. Rev Soc Bras Med Trop.

[B14] Weber SAT, Brasolotto A, Rodrigues L, Marcondes-Machado J, Padovani CR, Carvalho LR (2006). Dysphonia and laryngeal sequelae in paracoccidioidomycosis patients: a morphological and phoniatric study. Med Mycol.

[B15] Lopes JM, Severo LM, Mendes RP, Weber SAT (2011). Sequelae lesions in the larynxes of patients with Paracoccidioidomycosis. Braz J Otorhinolaryngol.

[B16] Bastos AGD, Martins AG, Cunha FC, Marques MPC, Melo PP, Tomita S (2001). Paracoccidioidomycosis of the Larynx: A 21 Year Review. Braz J Otorhinolaryngol.

[B17] Sant'anna GD, Mauri M, Arrarte JL, Camargo JRH (1999). Laryngeal Manifestations of Paracoccidioidomycosis (South American Blastomycosis). Arch Otolaryngol Head Neck Surg.

[B18] Fernandes PD, Fernandes LT (1986). Paracoccidioidomycosis. Rev Bras Otorrinolaringol.

[B19] Silva L, Damrose E, Bairão F, Della Nina ML, C J, Costa HO (2008). Infectious granulomatous laryngitis: a retrospective study of 24 cases. Eur Arch Otorhinolaryngol.

[B20] Machado J, Miranda JL, Teixeira GA (1965). On South American blastomycosis sequelae. Hospital.

[B21] Valle ACF, Aprigliano F, Moreira JS, Wanke B (1995). Clinical and endoscopic finding in the mucosae of the upper respiratory and digestive tracts in pos-treatment follow-up of paracoccidioidomycosis patients. Rev Inst Med Trop S Paulo.

[B22] da Costa AD, Vargas AP, Lucena MM, Ruas ACN, Braga FDSS, Bom-Braga MP (2017). Voice disorders in residual. paracoccidioidomycosis in upper airways and digestive tract. Rev Iberoam Micol.

[B23] Junqueira PAS, Trezza PM, Costa SS, Cruz OLM, Oliveira JAA (2006). Basic principles of voice therapy. Otorhinolaryngology: Principles and Practice.

[B24] Behlau M, Hogikyan ND, Gasparini G (2007). Quality of life and voice: study of a Brazilian population using the voice-related quality of life measure. Folia Phoniatr Logop.

[B25] Ugolino AC, Oliveira G, Behlau M (2012). Perceived dysphonia from the clinician’s and patient’s viewpoint. J Soc Bras Fonoaudiol.

[B26] Logemann JA (1983). Evaluation and Treatment of Swallowing Disorders.

[B27] Aviv JE, Murry T, Zschommler A, Cohen M, Gartner C (2005). Flexible endoscopic evaluation of swallowing with sensory testing: patient characteristics and analysis of safety in 1.340 consecutive examinations. Ann Otol Rhinol Laryngol.

[B28] Langmore SE (2003). Evaluation of oropharyngeal dysphagia: which diagnostic tool is superior?. Curr Opin Otolaryngol Head Neck Surg.

[B29] Queiroz MAS, Haguette RCB, Haguette EF (2009). Findings of fiberoptic endoscopy of swallowing in adults with neurogenic oropharyngeal dysphagia. Rev Soc Bras Fonaudiol.

[B30] Santoro P, Tsuji D, Lorenzi M, Ricci F (2003). The role of videoendoscopic swallowing study in the quantitative evaluation of the oral and pharyngeal phases of deglutition in the elderly. Arq Int Otorrinolaringol.

[B31] Seidl RO, Nusser-Müller-Busch R, Westhofen M, Ernst A (2008). Oropharyngeal findings of endoscopic examination in swallowing disorders of neurological origin. Eur Arch Otorhinolaryngol.

[B32] Tabaee A, Johnson PE, Gartner CJ, Kalwerisky K, Desloge RB, Stewart MG (2006). Patient-controlled comparison of flexible endoscopic evaluation of swallowing with sensory testing (FEESST) and videofluoroscopy. Laryngoscope.

